# The effect of mandatory post-call relief on sleep and wellness in ophthalmology residents

**DOI:** 10.1186/s12909-023-04947-1

**Published:** 2023-12-13

**Authors:** Shu Feng, John A. Davis, Apoorva Chowdhary, Whitney Lomazow, Jonathan S. Yi, Johnson Huang, Leona Ding, Parisa Taravati

**Affiliations:** 1 Department of Ophthalmology, University of Washington School of Medicine, Campus Box 359608, 325 9th Avenue, Seattle, WA 98104 USA; 2 Oregon Health and Sciences University, Casey Eye Institute, Portland, USA; 3 University of Miami Health System, Bascom Palmer Eye Institute, Miami, USA

**Keywords:** Post-call relief, Burnout, Ophthalmology, Residency, Sleep, Physical activity

## Abstract

**Background:**

Multiple duty hour reforms have been implemented to optimize resident wellness through increasing opportunities for sleep recovery, but few studies have recorded objectively measured sleep or shown direct sleep and wellness benefits from such interventions. This study seeks to determine whether mandatory post-call relief policies with a partial night float system improved resident sleep, activity, and burnout among ophthalmology residents taking home call.

**Methods:**

We conducted a two group cohort study of ophthalmology residents at the University Washington comparing post graduate year-2 (PGY-2) resident sleep, activity, and burnout between the optional post-call relief group from July 1, 2017 to June 30, 2019 to the mandatory post-call relief group from July 1, 2019 to June 30, 2021.

**Results:**

Of twenty total residents participating in the survey portion, 18 residents participated in the sleep and activity tracking portion of the study, 9 in in the optional post-call relief cohort, and 9 in the mandatory post-call relief cohort. The mandatory post-call relief group recorded longer total sleep on call than the optional post-call relief group (*p* < 0.001). There was no difference in overnight sleep recorded on call (median 3.4 h), but residents recorded more time napping in the mandatory post-call relief cohort (*p* < 0.001). There was no significant difference between cohorts in amount of sleep while not on call. Residents in the mandatory post-call relief cohort recorded higher average daily steps, higher exercise time, and lower sedentary time than residents in the optional post-call relief cohort (*p* < 0.001). They also recorded lower median emotional exhaustion on the Maslach Burnout Inventory and lower stress in the Depression and Anxiety Stress Scale in the mandatory post-call relief cohort (*p* = 0.008).

**Conclusions:**

Implementation of mandatory post-call relief policies with a partial night-float system among PGY-2 residents was associated with more post-call naps with more overall physical activity, lower emotional exhaustion scores, and lower stress scores, despite no changes to overnight sleep on call or total sleep. Although sample size limits interpretation of data, implementation of mandatory post call relief could be considered to improve post-call sleep in programs with home call.

## Background

Burnout is a work-related syndrome characterized by feelings of emotional exhaustion, depersonalization, and low sense of accomplishment. It has been shown to impair physician performance and increase medical errors [1] and up to 54% of physicians in the US report symptoms of burnout [2]. Work-related issues are not limited to attending physicians, and the Accreditation Council for Graduate Medical Education (ACGME) in its 2020 Common Program Requirements obligated residency programs to optimize resident well-being [3]. In studies of US resident physicians, up to 67% experience burnout [4], similar to the previously reported 63% among ophthalmology residents [5], with sleep deprivation and call duties among the most cited factors contributing to decreased wellbeing.

Numerous models describing the connection between sleep and burnout suggest either chronic depletion of energy stores or activation of the hypothalamic–pituitary–adrenal axis and increasing levels of bodily stress as a causative mechanism [6]. Multiple duty hour reforms have been implemented by the ACGME to address sleep deprivation, fatigue, and burnout among residents in the past two decades, and several studies have examined the effect of duty hour restrictions on resident well-being and sleep with mixed results [7–12]. Self-reported sleep deprivation has been shown to be associated with higher prevalence of moderate depression and clinical burnout among residents [10, 13, 14]. However, several studies have not found a significant association between burnout and sleep in resident physicians when sleep is measured through wrist actigraphy rather than subjective “sleepiness” [8, 9]. Interventions including night float, reduced shift length, and protected sleep time on call have not consistently produced improvements in resident wellness or sleep [15], but prior studies have not used objective sleep measures.

A prior study completed in the Department of Ophthalmology at the University of Washington studied objectively measured sleep on call using wrist-actigraphy, and showed evidence of resident burnout related to less sleep while on call [4]. Call polices were thus changed to mandate all post graduate year-2 (PGY-2) residents taking primary call to be excused from clinical duties starting at noon the following day. Residents on the research rotation were also scheduled for 2–3 sequential nights of call each week in a partial night-float model. Prior to this intervention, residents were on an every 5 night home-call system and had the option of leaving at noon the following day if they felt excessively tired, but this option was used variably. The goal of this study was to determine whether implementing mandatory post-call relief with partial night float for PGY-2 residents increased sleep and decreased resident burnout, depression, anxiety, and stress, using objectively measured sleep and activity measures and survey results.

## Methods

PGY-2 residents at the University of Washington Ophthalmology program cover primary weekday overnight calls (Monday through Friday 5 pm - 8am) and weekend calls (Saturday and Sunday 8am – 8am) for two hospitals, the Harborview Medical Center and University of Washington Medical Center. Residents took at-home call, such that they were not required to stay in-house.

Prior to the implementation of mandatory post-call relief (2017–2019), the five PGY-2 residents each year distributed calls equally among themselves and had an optional post-call relief system. If residents had clinical duties the following day, they could either continue to work through the next day or request post-call relief starting at noon the next day, which would be covered by a resident on a research rotation without assigned clinical duties for the day.

With the implementation of mandatory post-call relief (2019–2021), each of the 5 residents were assigned a call schedule based on their current rotation. A night float/research rotation was created, and the resident on this block was assigned 2–3 consecutive nights of call in a row (typically Sunday-Monday 8am – 8am, Monday 5 pm to 8am, and sometimes Tuesday 5 pm – 8am). The remaining four residents shared the 4–5 nights of call duties on a predictable schedule and were required to go home at noon on their post-call day, with no need for coverage of clinical duties in the afternoon. For example, when a resident was rotating on the oculoplastic rotation, he/she would take call every Wednesday night and not be given a clinical assignment Thursday afternoon, but when rotating on the neuro-ophthalmology rotation, he/she would take call every Thursday and not be given a clinical assignment on Friday afternoon. In the optional post-call relief system, taking post-call relief often required another resident to cover the post-call resident’s clinical duties the next day because the residents’ call schedule was variable.

Activity and sleep of PGY-2 ophthalmology residents before and after the implementation post-call relief policies were collected using Fitbit Alta HR wrist actigraphs (Fitbit, San Francisco, CA). Residents were asked to wear their Fitbits as much as tolerated throughout the entire PGY-2 year between July and June. Fitbit sleep and activity were matched to resident call schedules. Overnight sleep was defined as total “asleep” time recorded by the Fitbit device when the majority of the sleep period occurred between 8 pm and 8am. Sleep was considered a nap if the majority of the sleep period occurred between 8am and 8 pm. Recorded exercise was identified with Fitbit’s software classification of “Very Active Time.”

License to administer the Maslach Burnout Inventory was obtained (Mind Garden, Inc). All residents were asked to complete Maslach Burnout Inventory surveys (MBI) and Depression Anxiety Stress (DASS) five times throughout the year to assess burnout, once at the end of each 10–11-week clinical rotation. Median scores for each resident were used to compare cohorts.

Descriptive statistics and univariate analysis were performed with two-tailed t-test or Mann Whitney test using Prism 8 Software. Mixed model regression was conducted in R software to compare sleep and activity between optional and mandatory post-call relief groups.

## Results

Of twenty total residents participating in the survey portion, 18 residents participated in the Fitbit portion of the study, 9 in in the optional post-call relief cohort, and 9 in the mandatory post-call relief cohort. Table 1 illustrates demographics and data obtained from the two cohorts. Data obtained from each resident ranged from 12 to 330 days of activity data (median 141 days) and 2 to 289 days of sleep data (median 79).

**Table 1:** Demographics and data collection of optional and mandatory post-call relief cohorts

	Optional Post-Call Relief (2017–2019)	Mandatory Post-Call Relief (2019–2021)
Age (mean ± SD)	30 ± 2.3	29.3 ± 3.9
M:F ratio	4:6	7:3
# Nights recorded	791	1037
# On-call nights recorded	193	220
# Not on-call nights recorded	598	817
# Days of activity recorded	1590	1386

**Fig. 1:**
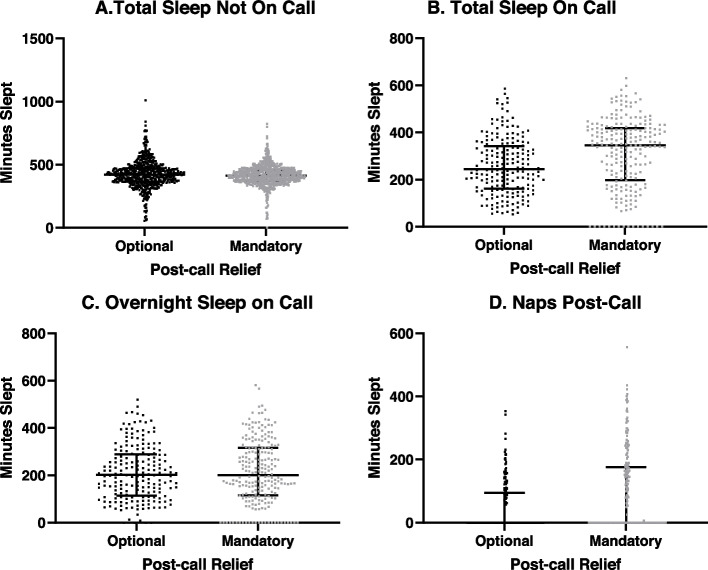
Aggregate sleep data comparing optional and mandatory post-call relief groups. In graphs **A** and **B**, each data point represents amount of sleep obtained over a single 24-h period while residents were **A**) Not On Call and **B**) On Call. Each data point in Graph **C** represents a sleep obtained overnight between 8 pm and 8am while on call. Each data point in graph **D** represents post-call sleep, in which the majority of sleep was obtained between 8am and 8pm following a night on call. Lines represent the median and interquartile range

Figure 1 compares aggregate sleep data among residents on call and not on call. Mixed model regression analysis was used to compare groups to adjust for the multiple measurements obtained from each subject. The mandatory post-call relief group recorded longer total sleep (overnight sleep + post-call naps) on call than the optional post-call relief group (*p* < 0.001). Median total on call sleep in the optional post call relief group was 4.0 h vs 5.8 h in the mandatory post-call relief group. There was no difference in overnight sleep on call, but residents recorded more time napping in the mandatory post-call relief cohort (*p* < 0.001). Median on-call overnight sleep in both the optional and mandatory post call relief groups were both 3.4 h, and median post-call nap duration was 0 for both groups. However, residents recorded naps only 34% of the time in the optional post-call relief group and 48% of the time in the mandatory post call relief group. The mandatory post call relief group also recorded longer naps, with mean 3.4 ± 1.7 h compared to 2.4 ± 1.1 h in the optional post-call relief group. There was no significant difference between cohorts in amount of sleep not while on call (*p* = 0.11), as median sleep while not on call was 6.9 h in the optional post call relief group and 7.1 h in the mandatory post call relief group.

**Fig. 2:**
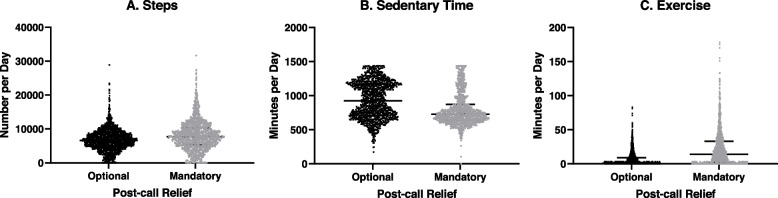
Aggregate activity data comparing option and mandatory post-call relief groups. Each data point represents a recorded steps, sedentary time, and exercise during single day of activity with lines representing median and interquartile range

Aggregate activity data is compared between cohorts in Fig. 2. Residents in the mandatory post-call relief cohort recorded higher average daily steps, higher exercise time, and lower sedentary time than residents in the optional post-call relief cohort (*p* < 0.001).

**Fig. 3:**
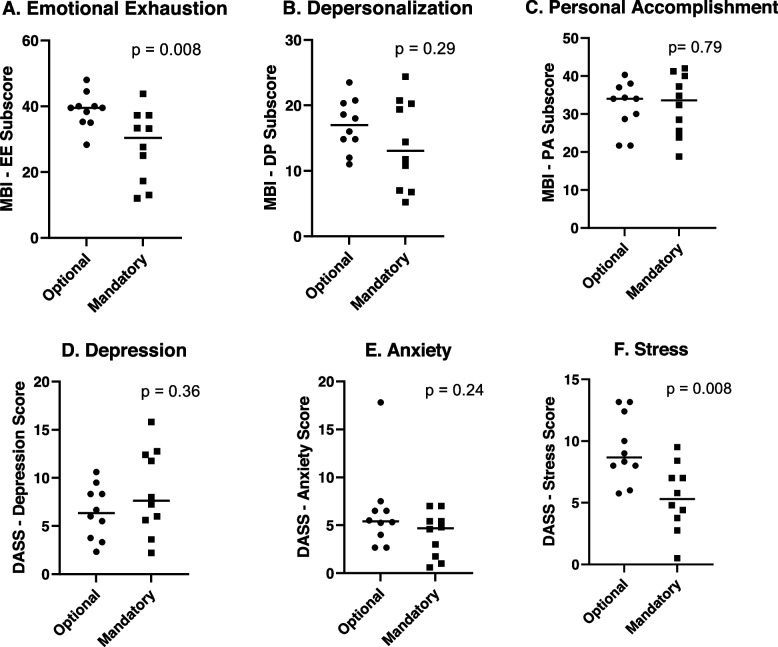
Median Maslach Burnout Inventory (MBI) and Depression, Anxiety, and Stress Scale (DASS) subscores for each resident are represented by individual data points, with lines presenting median score for each group. *P*-values from the Mann–Whitney test comparing optional and mandatory post-call relief groups are listed for each graph

Maslach Burnout Inventory and Depression, Anxiety, and Stress scores before and after the intervention are shown in Fig. 3. There was significantly lower median emotional exhaustion on the Maslach Burnout Inventory in the mandatory post-call relief cohort (*p* = 0.008), but no significant difference in median depersonalization and personal accomplishment scores. There was also a significantly lower median stress score (*p* = 0.008) in the mandatory post-call relief group but no significant difference in depression and anxiety scores on the Depression, Anxiety, and Stress Scale.

## Discussion

ACGME requirements regarding call primarily address in-house call, which is subject to a limitation of no more than 30 h of continuous on-site duty. However, ophthalmology call, as well as call in smaller specialty programs, is frequently “at-home call,” with ACGME requirements specifying only that it must be “not so frequent or taxing as to preclude rest or reasonable personal time for each resident.” We sought to identify whether changing call structure for “at-home call” to allow for sleep recovery would improve resident sleep and burnout and found that implementation of mandatory post-call relief policies with a partial night-float system among PGY-2 residents in our program were associated with more post-call naps, more physical activity, lower emotional exhaustion, and lower stress, despite no changes to overnight sleep on call or total sleep.

In the complex relationships between sleep, activity, and wellness, the greater protected time away from work may have allowed burnout recovery through protected time for sleep and other recovery measures. Data suggests that protected time away from work can lead to restoration of sleep quality and reduced long-term stress [16]. Work hour limitations have generally been reported to have positive effects on resident well-being [13, 17], but interventions including night float, reduced shift length, and protected sleep time on call have not consistently produced improvements in resident wellness, patient care, or resident education [15]. In our case, while we found significant improvement of emotional exhaustion and stress, we found no significant difference among cohorts in depression, anxiety, depersonalization, or personal accomplishment scores. While changing call structure to improve sleep, and activity may be overall beneficial, that these other aspects of wellbeing were unchanged speaks to the multifaceted nature of wellness and might explain why many prior studies have not found improvement in resident wellness with interventions targeting sleep deprivation [15].

While it is assumed that recovery of sleep is a primary driver in improving well-being in our cohort, residents’ time allocation when post call has been noted to be dedicated both to recovery of sleep and to recovery of self, including reconnecting with identities, roles, and relationships outside of medicine [18]. In our study, residents still napped just 48% of post-call days, suggesting that some of the post-call recovery time was dedicated to other aspects of recovery and self-care. One such aspect of recovery is physical activity. At baseline, residents are less likely than medical students and attending physicians to engage in physical activity [19], and this lack of physical activity could contribute to burnout. One study found internal medicine residents exercising at least 150 min per week were less likely to experience burnout, and that those residents who cited “lack of time” and “lack of energy” as reasons for foregoing physical activity were much more likely to experience burnout [20]. The increase in sleep and protected recovery time in PGY-2 residents could be contributing to their ability to partake in physical activity, improving both their physical and mental well-being.

This study’s non-randomized two-group design with data obtained from a single ophthalmology program with a small sample size limits interpretation of data. There was also variable adherence among individuals to Fitbit usage, and individual differences in sleep, activity, and wellness cannot be accounted for by call structure alone. Other factors, such as the Covid 19 pandemic may have affected results, as volume of call may have decreased during the pandemic [21]. Additionally, other program changes such as changes to clinical schedules could have influenced results. Despite these limitations, the aggregate collection of over 1800 nights of sleep data and 2900 days of activity data via Fitbits in our cohort allows for unique insight into resident sleep and activity patterns. While this study’s findings may not be generalizable to other programs, it proposes mandatory post call relief as a method to improve post-call sleep, as well as illustrates a method in which other programs can study interventions targeted toward improving resident sleep and wellness. Future studies could study similar interventions in a larger sample size across multiple institutions.

## Conclusion

The ideal structure for overnight call for residents is likely highly dependent on specific program characteristics and individual resident sleep preferences. However, increasing protected time post-call to allow for sleep recovery may be a beneficial intervention to reduce resident burnout. Our data illustrates that implementation of mandatory post-call relief with a partial night float system is one mechanism that may be associated with improvement in resident sleep on call, specifically through increasing naps taken post-call. Despite not changing overall sleep or overnight sleep on call, these changes allowed for dedicated recovery time with increased physical activity, decreased sedentary time, and lower emotional exhaustion and stress among PGY-2 ophthalmology residents in this program. Programs seeking to implement changes to call structure can similarly use sleep and activity tracking in combination with surveys to study the impact of such interventions.

## Data Availability

The datasets used and/or analyzed during the current study are available from the corresponding author on reasonable request.
